# Prevalence and Characteristics of Autism Spectrum Disorder Among Children Aged 8 Years — Autism and Developmental Disabilities Monitoring Network, 11 Sites, United States, 2020

**DOI:** 10.15585/mmwr.ss7202a1

**Published:** 2023-03-24

**Authors:** Matthew J. Maenner, Zachary Warren, Ashley Robinson Williams, Esther Amoakohene, Amanda V. Bakian, Deborah A. Bilder, Maureen S. Durkin, Robert T. Fitzgerald, Sarah M. Furnier, Michelle M. Hughes, Christine M. Ladd-Acosta, Dedria McArthur, Elise T. Pas, Angelica Salinas, Alison Vehorn, Susan Williams, Amy Esler, Andrea Grzybowski, Jennifer Hall-Lande, Ruby H.N. Nguyen, Karen Pierce, Walter Zahorodny, Allison Hudson, Libby Hallas, Kristen Clancy Mancilla, Mary Patrick, Josephine Shenouda, Kate Sidwell, Monica DiRienzo, Johanna Gutierrez, Margaret H. Spivey, Maya Lopez, Sydney Pettygrove, Yvette D. Schwenk, Anita Washington, Kelly A. Shaw

**Affiliations:** ^1^National Center on Birth Defects and Developmental Disabilities, CDC, Atlanta, Georgia; ^2^Vanderbilt University Medical Center, Nashville, Tennessee; ^3^Oak Ridge Institute for Research and Education, Oak Ridge, Tennessee; ^4^University of Utah School of Medicine, Salt Lake City, Utah; ^5^University of Wisconsin, Madison, Wisconsin; ^6^Washington University, St. Louis, Missouri; ^7^Johns Hopkins Bloomberg School of Public Health, Baltimore, Maryland; ^8^University of Minnesota, Minneapolis, Minnesota; ^9^University of California, San Diego, California; ^10^Rutgers New Jersey Medical School, Newark, New Jersey; ^11^University of Arkansas for Medical Sciences, Little Rock, Arkansas; ^12^University of Arizona, Tucson, Arizona

## Abstract

**Problem/Condition:**

Autism spectrum disorder (ASD).

**Period Covered:**

2020.

**Description of System:**

The Autism and Developmental Disabilities Monitoring (ADDM) Network is an active surveillance program that provides estimates of the prevalence of ASD among children aged 8 years. In 2020, there were 11 ADDM Network sites across the United States (Arizona, Arkansas, California, Georgia, Maryland, Minnesota, Missouri, New Jersey, Tennessee, Utah, and Wisconsin). To ascertain ASD among children aged 8 years, ADDM Network staff review and abstract developmental evaluations and records from community medical and educational service providers. A child met the case definition if their record documented 1) an ASD diagnostic statement in an evaluation, 2) a classification of ASD in special education, or 3) an ASD *International Classification of Diseases* (ICD) code.

**Results:**

For 2020, across all 11 ADDM sites, ASD prevalence per 1,000 children aged 8 years ranged from 23.1 in Maryland to 44.9 in California. The overall ASD prevalence was 27.6 per 1,000 (one in 36) children aged 8 years and was 3.8 times as prevalent among boys as among girls (43.0 versus 11.4). Overall, ASD prevalence was lower among non-Hispanic White children (24.3) and children of two or more races (22.9) than among non-Hispanic Black or African American (Black), Hispanic, and non-Hispanic Asian or Pacific Islander (A/PI) children (29.3, 31.6, and 33.4 respectively). ASD prevalence among non-Hispanic American Indian or Alaska Native (AI/AN) children (26.5) was similar to that of other racial and ethnic groups. ASD prevalence was associated with lower household income at three sites, with no association at the other sites.

Across sites, the ASD prevalence per 1,000 children aged 8 years based exclusively on documented ASD diagnostic statements was 20.6 (range = 17.1 in Wisconsin to 35.4 in California). Of the 6,245 children who met the ASD case definition, 74.7% had a documented diagnostic statement of ASD, 65.2% had a documented ASD special education classification, 71.6% had a documented ASD ICD code, and 37.4% had all three types of ASD indicators. The median age of earliest known ASD diagnosis was 49 months and ranged from 36 months in California to 59 months in Minnesota.

Among the 4,165 (66.7%) children with ASD with information on cognitive ability, 37.9% were classified as having an intellectual disability. Intellectual disability was present among 50.8% of Black, 41.5% of A/PI, 37.8% of two or more races, 34.9% of Hispanic, 34.8% of AI/AN, and 31.8% of White children with ASD. Overall, children with intellectual disability had earlier median ages of ASD diagnosis (43 months) than those without intellectual disability (53 months).

**Interpretation:**

For 2020, one in 36 children aged 8 years (approximately 4% of boys and 1% of girls) was estimated to have ASD. These estimates are higher than previous ADDM Network estimates during 2000–2018. For the first time among children aged 8 years, the prevalence of ASD was lower among White children than among other racial and ethnic groups, reversing the direction of racial and ethnic differences in ASD prevalence observed in the past. Black children with ASD were still more likely than White children with ASD to have a co-occurring intellectual disability.

**Public Health Action:**

The continued increase among children identified with ASD, particularly among non-White children and girls, highlights the need for enhanced infrastructure to provide equitable diagnostic, treatment, and support services for all children with ASD. Similar to previous reporting periods, findings varied considerably across network sites, indicating the need for additional research to understand the nature of such differences and potentially apply successful identification strategies across states.

## Introduction

Autism spectrum disorder (ASD) is a developmental disability characterized by persistent impairments in social interaction and the presence of restricted, repetitive patterns of behaviors, interests, or activities (*1*) that can cause a wide array of difficulties in social interaction, communication, and participation in daily activities. CDC began monitoring the prevalence of ASD in metropolitan Atlanta, Georgia, in 1996 as part of its Metropolitan Atlanta Developmental Disabilities Surveillance Program (*2*). CDC established the Autism and Developmental Disabilities Monitoring (ADDM) Network in 2000 and used the model developed in metropolitan Atlanta to track ASD prevalence in additional areas of the country. Starting with the 2000 surveillance year, the ADDM Network has reported ASD prevalence for even-numbered years (*3*–*12*). This is the 11th surveillance summary published in *MMWR* and marks a period of 20 years of monitoring ASD in multiple U.S. communities.

During the past two decades, ASD prevalence estimates of children aged 8 years from the ADDM Network have increased markedly, from 6.7 (one in 150) per 1,000 in 2000 to 23.0 (one in 44) in 2018 (*3*,*12*). In addition, overall ASD prevalence among White children was 50% higher than among Black or African American (Black) or Hispanic children in earlier years. (Persons of Hispanic origin might be of any race but are categorized as Hispanic; all racial groups are non-Hispanic). These gaps narrowed over time until ASD prevalence among Black and Hispanic matched prevalence among White children for the first time in 2016 and 2018, respectively (*11*,*12*). Similarly, robust associations between autism prevalence and higher socioeconomic status were observed in ADDM Network sites during 2002–2010 (*13*); however, this association was much more variable in 2018 (*12*). These patterns have largely been interpreted as improvements in more equitable identification of ASD, particularly for children in groups that have less access or face greater barriers in obtaining services (including diagnostic evaluations). However, consistent disparities for co-occurring intellectual disability exist because among all children with ASD, Black children have the largest proportion identified with intellectual disability (*10*–*12*).

This report describes ASD prevalence and characteristics among children aged 8 years from 11 ADDM Network sites in 2020, including prevalence by site and demographic characteristics, median ages when children with ASD were first evaluated or identified, and the co-occurrence of intellectual disability. These data can be used by service providers, educators, communities, researchers, and policymakers to track trends and support efforts to ensure the equitable allocation of needed services and support for all children with ASD.

## Methods

### Surveillance Sites and Procedures

For 2020, the ADDM Network included 11 sites (Arizona, Arkansas, California, Georgia, Maryland, Minnesota, Missouri, New Jersey, Tennessee, Utah, and Wisconsin) that monitored ASD prevalence. Each site selected a geographic area of its state to monitor ASD among children aged 8 years (Table 1). Children included in this report were born in 2012 and lived in surveillance areas of the 11 sites during 2020. Sites were competitively funded and functioned as public health authorities under the Health Insurance Portability and Accountability Act of 1996 Privacy Rule and met applicable local institutional review board, privacy, and confidentiality requirements under 45 CFR 46 (*14*).

**TABLE 1:** Surveillance sites and data sources used for surveillance in each site — Autism and Developmental Disabilities Monitoring Network, 11 sites, United States, 2020

Site	Surveillance area description	Total population aged 8 yrs	% American Indian or Alaska Native*	% Asian or Pacific Islander	% Black	% Hispanic	% White	% Two or more races	Types of data sources used^†^	Education data sources (% population coverage)^§^	% of requested records fully accessible for chart review
Arizona	Part of one county in metropolitan Phoenix	13,118^¶^	3.1	2.9	6.8	41.8	40.3	5.1	Health, education, Medicaid	100	100
Arkansas	21 counties in central Arkansas	15,432	0.3	1.3	24.2	9.1	60.8	4.2	Health, education	100	100
California	Part of one county in metropolitan San Diego	15,828^¶^	0.3	11.9	7.1	49.4	23.1	8.3	Health, education, state developmental disability services	100	100
Georgia	Two counties in metropolitan Atlanta	21,921	0.1	7.4	51.1	11.8	25.7	3.9	Health, education	97.6	85.9
Maryland	Five counties in suburban Baltimore	21,278	0.2	9.5	23.9	9.0	51.2	6.1	Health, education, early intervention	100	71.5
Minnesota	Parts of three counties in the Twin Cities metropolitan area	16,150^¶^	1.1	16.3	23.3	10.9	41.8	6.6	Health, education	100	100
Missouri	Five counties in metropolitan St. Louis	24,561	0.1	3.4	23.8	4.8	63.0	4.8	Health, education	50.3	99.9
New Jersey	Two counties in New York metropolitan area	18,940	0.2	6.3	30.5	33.6	26.6	2.8	Health, education	100	95.8
Tennessee	11 counties in middle Tennessee	25,588	0.2	3.4	17.2	13.5	60.4	5.3	Health, education	100	66.3
Utah	Three counties in northern Utah	24,734	0.6	4.2	1.8	20.7	68.4	4.2	Health, education, early intervention	100	87.6
Wisconsin	Eight counties in southeastern Wisconsin	28,789	0.3	5.5	17.0	17.4	54.8	5.0	Health, education, early intervention, Medicaid claims, state-funded long-term care program	100	100
**Total**		**226,339**	**0.5**	**6.3**	**20.8**	**18.5**	**48.7**	**5.1**	—	**99.9**	**91.8**

* Persons of Hispanic origin might be of any race but are categorized as Hispanic; all racial groups are non-Hispanic.

^†^ Health sources include records from medical and service providers that evaluate children with developmental disabilities.

^§^ For public schools in the surveillance area. In the absence of direct access to education sources, education data could be collected if they were included in a child’s medical or service records.

^¶^ Denominator excludes school districts that were not included in the surveillance area, calculated from National Center for Education Statistics enrollment counts of third graders during the 2020–21 school year.

### Case Ascertainment and Surveillance Case Definition

The ADDM Network conducts active surveillance of ASD by using multiple sources of information within a community (Table 1). The methods for collecting information and the case definition were unchanged from the 2018 surveillance year (*12*) and were modeled after those developed by CDC’s Metropolitan Atlanta Developmental Disabilities Surveillance Program (*3*). Sites request records from community medical, education, and service providers containing specific billing codes from the *International Classification of Diseases, Ninth Revision* (ICD-9) or *International Classification of Diseases, Tenth Revision* (ICD-10) or special education classification. The protocol allowed each site to select the ICD codes that necessitate record review if those codes closely aligned with program-recommended ICD codes (*11*). All ADDM Network sites used records from medical service providers that evaluated children with developmental disabilities and, for the first time, all sites had at least partial access to public school education records (Table 1). ADDM Network sites received information (including demographic data and ICD codes or special education classifications) for children with one or more of the requested codes or classifications, and ADDM staff manually reviewed the contents of associated (electronic and paper-based) records when available. If any part of the child’s record contained information meeting the case definition, ADDM staff abstracted information from the child’s developmental evaluations, special education plans, and other documents (e.g., cognitive or IQ tests) from all data sources. At certain sites, full record review could not be completed for all records because of the COVID-19 pandemic or other restrictions on physically accessing the location where records were stored (Table 1).

Children met the ASD case definition if they were aged 8 years in 2020 (born in 2012), lived in the surveillance area for at least 1 day during 2020, and had documentation in their records that they ever received 1) a written ASD diagnostic statement from a qualified professional, 2) a special education classification of autism (either primary exceptionality of ASD or an evaluation reporting criterion for autism eligibility was met) in public school, or 3) an ASD ICD code (ICD-9 codes between 299.00 and 299.99 or ICD-10 codes in the F84 range except for F84.2, Rett syndrome) obtained from administrative or billing information. Five children had an ICD code for Rett syndrome (F84.2) and no other indicators of ASD and did not meet the ASD case definition. ASD-related diagnostic conclusions (including suspected ASD or ruled out ASD) were collected verbatim from evaluations and were reviewed and classified by ADDM Network staff with clinical expertise at each site.

### Additional Data Sources and Variable Definitions

Population denominators were obtained from the U.S. Census vintage 2021 county-level single-year-of-age postcensal population estimates for 2020 (*15*). In this report, the Asian and Native Hawaiian or other Pacific Islander categories were combined into Asian or Pacific Islander because current systems often combine these categories or are not explicit whether “Asian” at a given data source includes “Native Hawaiian or other Pacific Islander.” Population denominators include categories for American Indian or Alaska Native (AI/AN), Asian or Pacific Islander (A/PI), Black, White, two or more races, and Hispanic ethnicity. In previous ADDM Network reports, the denominator data were based on the National Center for Health Statistics postcensal bridged race estimates (also produced by the Census Bureau) (*16*); the bridged race data set did not include a category for two or more races, which increased counts in the other categories.

Surveillance areas at three sites (Arizona, California, and Minnesota) comprised subcounty school districts. For these sites, county population estimates were adjusted using the National Center for Education Statistics public school enrollment counts and the American Community Survey tract-level ages 5–9 years population estimates described previously (*12*). The primary race and ethnicity and sex information came from medical or education records and, when missing, was augmented by birth certificate linkages (among children born in the state of their residence at age 8 years), administrative, or billing information. Children with missing or unknown race or ethnicity information were excluded from race- and ethnicity-specific prevalence estimates.

Census tract-level median household income (MHI) was measured using the 2020 American Community Survey 5-year estimates (*17*). Population counts of children aged 8 years were estimated by dividing the number of children aged 5–9 years by five for each census tract. The tracts included in the surveillance areas were classified into three approximately equal-sized population groups (i.e., tertiles) of low, medium, and high MHI by using data from all sites. Children meeting the ADDM Network case definition for ASD were geocoded and assigned to a socioeconomic status (SES) group corresponding to their 2020 address. Census tract information was available for 96.0% of children; the remainder could not be linked to a census tract but had service receipt or school attendance that indicated study area residence.

A child was classified as having intellectual disability if they had an IQ score ≤70 on their most recent cognitive test or intellectual disability was indicated in a statement in a developmental evaluation from a qualified professional. Children were classified in the borderline range for IQ if the score on their most recent test was 71–85, and in the average or higher range with most recent IQ score >85 or with a statement their IQ was in the average range without a specific score. Age at first developmental evaluation was limited to children with information on the earliest collected or historically reported evaluation. Age at first ASD diagnosis was based on the earliest documented age when a qualified professional diagnosed ASD.

### Analytic Methods

Prevalence was calculated as the number of children with ASD per 1,000 children in the defined population or group. Overall ASD prevalence estimates included all children with ASD from all 11 sites. Prevalence also was stratified by sex and by race and ethnicity using both the U.S. Census postcensal population estimates as well as the National Center for Health Statistics postcensal bridged race denominators. The Wilson score method was used to calculate 95% CIs. Pearson chi-square tests were used to compare proportions, and the Mantel-Haenszel (Woolf) test of homogeneity compared prevalence ratios across sites. Permutation tests were conducted to test differences in medians. Cochran Armitage tests were used to detect trends in prevalence across SES tertiles. Prevalence estimates with a relative SE >30% (and ratios calculated from those estimates) were considered to have limited statistical precision and were suppressed. Statistical tests with p values <0.05 and prevalence ratio 95% CIs that excluded 1.0 were considered statistically significant. R software (version 4.2; R Foundation) and additional packages were used to conduct analyses (*12*).

## Results

### ASD Prevalence

The overall ASD prevalence per 1,000 children aged 8 years was 27.6 (one in 36) and ranged from 23.1 in Maryland to 44.9 in California (Table 2). The overall male-to-female prevalence ratio was 3.8, with overall ASD prevalence of 43.0 among boys and 11.4 among girls. The same sites conducted ASD surveillance in 2018 and reported a combined prevalence of 23.0; however, certain sites changed their geographic areas or access to data sources for the current reporting period (Supplementary Table 1, https://stacks.cdc.gov/view/cdc/124397). The two sites with the largest relative changes (Missouri [48.5%] and Wisconsin [49.5%]) from 2018 to 2020 had increased access to education records in 2020 but no change in the geographic areas.

**TABLE 2:** Prevalence* of autism spectrum disorder among children aged 8 years, overall and by sex — Autism and Developmental Disabilities Monitoring Network, 11 sites, United States, 2020

Site	Overall^†^			Male prevalence (95% CI)	Female prevalence (95% CI)	Male-to-female prevalence ratio (95% CI)^§^
	No. with ASD	Total population	Prevalence (95% CI)			
Arizona	360	13,118	27.4 (24.8–30.4)	43.8 (39.2–49.0)	10.3 (8.1–13.1)	4.3 (3.3–5.5)
Arkansas	362	15,432	23.5 (21.2–26.0)	36.3 (32.4–40.6)	9.6 (7.6–12.1)	3.8 (2.9–4.9)
California	710	15,828	44.9 (41.7–48.2)	69.4 (64.1–75.1)	19.1 (16.3–22.4)	3.6 (3.0–4.3)
Georgia	553	21,921	25.2 (23.2–27.4)	40.2 (36.7–44.0)	9.7 (8.0–11.7)	4.2 (3.4–5.1)
Maryland	491	21,278	23.1 (21.1–25.2)	36.9 (33.5–40.6)	8.6 (7.0–10.6)	4.3 (3.4–5.4)
Minnesota	482	16,150	29.8 (27.3–32.6)	47.8 (43.4–52.6)	11.0 (9.0–13.6)	4.3 (3.4–5.4)
Missouri	601	24,561	24.5 (22.6–26.5)	38.7 (35.4–42.2)	9.3 (7.8–11.2)	4.1 (3.4–5.1)
New Jersey	544	18,940	28.7 (26.4–31.2)	44.5 (40.5–48.7)	12.2 (10.2–14.7)	3.6 (3.0–4.5)
Tennessee	713	25,588	27.9 (25.9–30.0)	43.9 (40.5–47.5)	11.1 (9.4–13.1)	4.0 (3.3–4.8)
Utah	621	24,734	25.1 (23.2–27.1)	37.6 (34.4–41.1)	11.8 (10.0–13.9)	3.2 (2.6–3.8)
Wisconsin	808	28,789	28.1 (26.2–30.0)	42.6 (39.4–45.9)	13.0 (11.2–15.0)	3.3 (2.8–3.9)
**Total**	**6,245**	**226,339**	**27.6 (26.9–28.3)**	**43.0 (41.9–44.2)**	**11.4 (10.7–12.0)**	**3.8 (3.6–4.0)**

**Abbreviation:** ASD = autism spectrum disorder.

* Per 1,000 children aged 8 years.

^†^ All children are included in the total regardless of sex or race and ethnicity.

^§^ Wilson score 95% CIs exclude 1.0 in all sites, indicating significantly higher prevalence among males than among females; Mantel Haenszel (Woolf) test of homogeneity of prevalence ratios across sites, p value = 0.15, indicating little heterogeneity in prevalence ratios across sites.

Overall, ASD prevalence per 1,000 children aged 8 years differed by racial and ethnic groups (Table 3); prevalence among White children (24.3) was lower than prevalence among Black, Hispanic, or A/PI children (29.3, 31.6, and 33.4, respectively). Among AI/AN children, ASD prevalence was 26.5 overall and was similar to other groups, but estimates met the 30% relative SE threshold for statistical precision in just one site (Arizona). ASD prevalence among children of two or more races was 22.9, which was not different than among White children but was lower than prevalence among AP/I, Black, and Hispanic children. Missouri was the only site in which White children had higher ASD prevalence than another racial or ethnic group (White compared with two or more races). Additional prevalence ratios comparing racial and ethnic groups are available (Supplementary Table 2, https://stacks.cdc.gov/view/cdc/124397). Prevalence calculations using the bridged-race denominator racial and ethnic categories used in previous reports (Supplementary Table 3, https://stacks.cdc.gov/view/cdc/124397) yielded similar findings of lower ASD prevalence among White children compared with that among Asian, Black, and Hispanic children.

**TABLE 3:** Prevalence* of autism spectrum disorder among children aged 8 years, by race and ethnicity^†^ — Autism and Developmental Disabilities Monitoring Network, 11 sites, United States, 2020

Site	Prevalence (95% CI)					Prevalence Ratio (95% CI)			
	A/PI	Black	Hispanic	White	Two or more races	Black to White	Hispanic to White	A/PI to White	Two or more races to White
Arizona	—^§^	25.9 (17.3–38.6)	26.6 (22.7–31.2)	29.7 (25.5–34.7)	20.9 (12.5–34.8)	0.9 (0.6–1.3)	0.9 (0.7–1.1)	—	0.7 (0.4–1.2)
Arkansas	58.8 (34.0–100.0)	23.9 (19.4–29.3)	31.0 (23.2–41.4)	22.5 (19.7–25.7)	—	1.1 (0.8–1.4)	1.4 (1.0–1.9)^¶^	2.6 (1.5–4.6)^¶^	—
California	56.5 (46.9–67.9)	44.4 (33.8–58.1)	45.3 (40.9–50.1)	38.3 (32.6–45.1)	39.7 (30.4–51.7)	1.2 (0.8–1.6)	1.2 (1.0–1.4)	1.5 (1.2–1.9)^¶^	1.0 (0.8–1.4)
Georgia	25.3 (18.7–34.1)	28.6 (25.7–31.8)	25.2 (19.8–32.0)	19.0 (15.7–22.9)	17.6 (10.7–28.9)	1.5 (1.2–1.9)^¶^	1.3 (1.0–1.8)	1.3 (0.9–1.9)	0.9 (0.5–1.6)
Maryland	36.5 (29.2–45.6)	33.6 (29.0–39.0)	17.2 (12.2–24.0)	16.8 (14.5–19.4)	19.1 (13.0–28.1)	2.0 (1.6–2.5)^¶^	1.0 (0.7–1.5)	2.2 (1.7–2.8)^¶^	1.1 (0.8–1.7)
Minnesota	24.3 (19.1–30.9)	27.9 (23.1–33.7)	40.4 (32.2–50.7)	30.0 (26.2–34.4)	31.0 (22.1–43.2)	0.9 (0.7–1.2)	1.3 (1.0–1.8)^¶^	0.8 (0.6–1.1)	1.0 (0.7–1.5)
Missouri	34.3 (24.0–48.9)	28.1 (24.2–32.7)	16.8 (10.9–25.8)	23.4 (21.1–25.9)	10.2 (5.8–7.7)	1.2 (1.0–1.4)^¶^	0.7 (0.5–1.1)	1.5 (1.0–2.1)^¶^	0.4 (0.2–0.8)^¶^
New Jersey	27.5 (19.6–38.3)	32.9 (28.6–37.8)	32.7 (28.6–37.3)	19.7 (16.2–23.9)	—	1.7 (1.3–2.1)^¶^	1.7 (1.3–2.1)^¶^	1.4 (0.9–2.1)	—
Tennessee	38.3 (27.4–53.3)	32.9 (28.0–38.6)	26.3 (21.5–32.2)	25.2 (22.9–27.8)	25.7 (18.5–35.6)	1.3 (1.1–1.6)^¶^	1.0 (0.8–1.3)	1.5 (1.1–2.2)^¶^	1.0 (0.7–1.4)
Utah	27.9 (19.5–39.8)	—	23.6 (19.8–28.1)	24.8 (22.5–27.2)	18.2 (11.7–28.3)	—	1.0 (0.8–1.2)	1.1 (0.8–1.6)	0.7 (0.5–1.2)
Wisconsin	29.2 (22.0–38.7)	23.8 (19.9–28.5)	35.6 (30.8–41.1)	25.9 (23.5–28.5)	30.0 (22.4–40.2)	0.9 (0.8–1.1)	1.4 (1.2–1.6)^¶^	1.1 (0.8–1.5)	1.2 (0.9–1.6)
**Total**	**33.4 (30.5–36.4)**	**29.3 (27.9–30.9)**	**31.6 (30.0–33.3)**	**24.3 (23.4–25.2)**	**22.9 (20.3–25.8)**	**1.2 (1.1–1.3)** ^¶^	**1.3 (1.2–1.4)** ^¶^	**1.4 (1.2–1.5)** ^¶^	**0.9 (0.8–1.1)**

**Abbreviation:** A/PI = Asian or Pacific Islander.

* Per 1,000 children aged 8 years. Overall American Indian/Alaska Native autism spectrum disorder prevalence per 1,000 was 26.5 (95% CI = 18.5–37.8). Arizona was the only Autism and Developmental Disabilities Monitoring Network site meeting the threshold for statistical precision for American Indian/Alaska Native autism spectrum disorder prevalence; the site-specific prevalence per 1,000 was 26.8 (95% CI = 15.0–47.3). None of ratios with AI/AN children that met the threshold for suppression were statistically significant.

^†^ Persons of Hispanic origin might be of any race but are categorized as Hispanic; all racial groups are non-Hispanic.

^§^ Dash indicates estimate was suppressed because SE for prevalence was ≥30% of estimate, or prevalence ratio was based on an estimate that was suppressed.

^¶^ 95% CI does not include 1.0.

**Figure 1:**
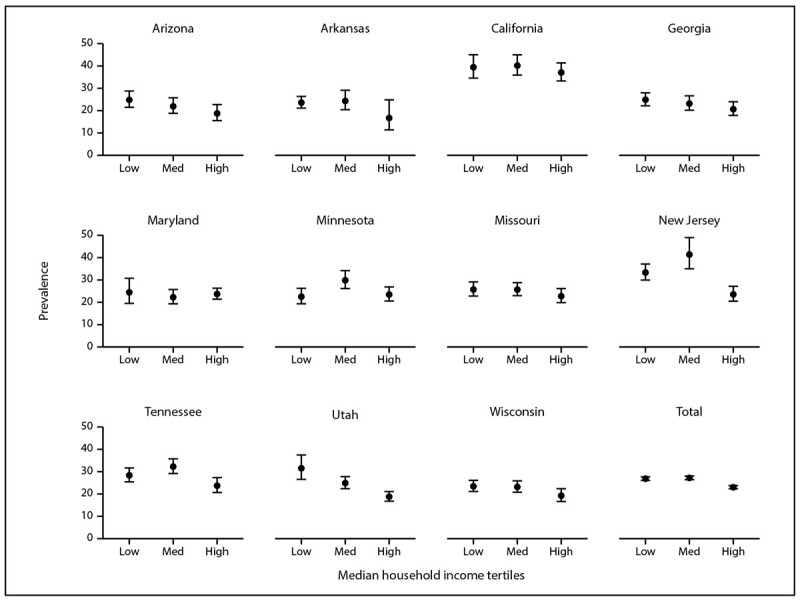
Prevalence* of autism spectrum disorder among children aged 8 years, by median household income tertile and site — Autism and Developmental Disabilities Monitoring Network, 11 sites, United States, 2020^†^ * Per 1,000 children aged 8 years. Dots are the point estimates and horizontal lines are the 95% CIs. ^†^ Cochran Armitage test of trend for association between socioeconomic status tertile and ASD prevalence, by site and overall: Arizona p = 0.03; Arkansas p = 0.3; California p = 0.5; Georgia p = 0.08; Maryland p = 0.9; Minnesota p = 0.8; Missouri p = 0.3; New Jersey p<0.01; Tennessee p = 0.1; Utah p<0.01; Wisconsin p = 0.08; Total p<0.01.

In eight sites, ASD prevalence was not associated with census tract-level MHI, but in three sites (Arizona, New Jersey, and Utah), lower ASD prevalence was observed among children living in census tracts with higher MHI (Figure 1). When all sites were combined, prevalence of ASD was lower among census tracts with higher MHI; however, ASD prevalences for the low, medium, and high SES tertiles were all between 23.0–27.2.

### ASD Identification

**Figure 2:**
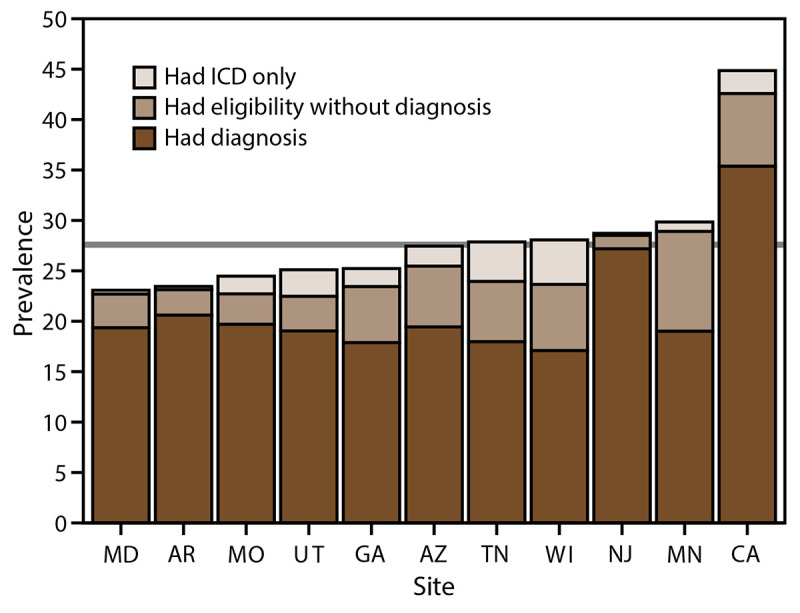
Prevalence* of autism spectrum disorder among children aged 8 years, by identification type and site — Autism and Developmental Disabilities Monitoring Network, 11 sites, United States, 2020^†^ **Abbreviation:** ICD = International Classification of Diseases. *Per 1,000 children aged 8 years. ^†^ Horizontal line is the overall Autism and Developmental Disabilities Monitoring Network prevalence of 27.6 per 1,000 children aged 8 years. Children with documented autism spectrum disorder (ASD) statements could also have ASD classifications in special education or ASD ICD codes.

**Figure 3:**
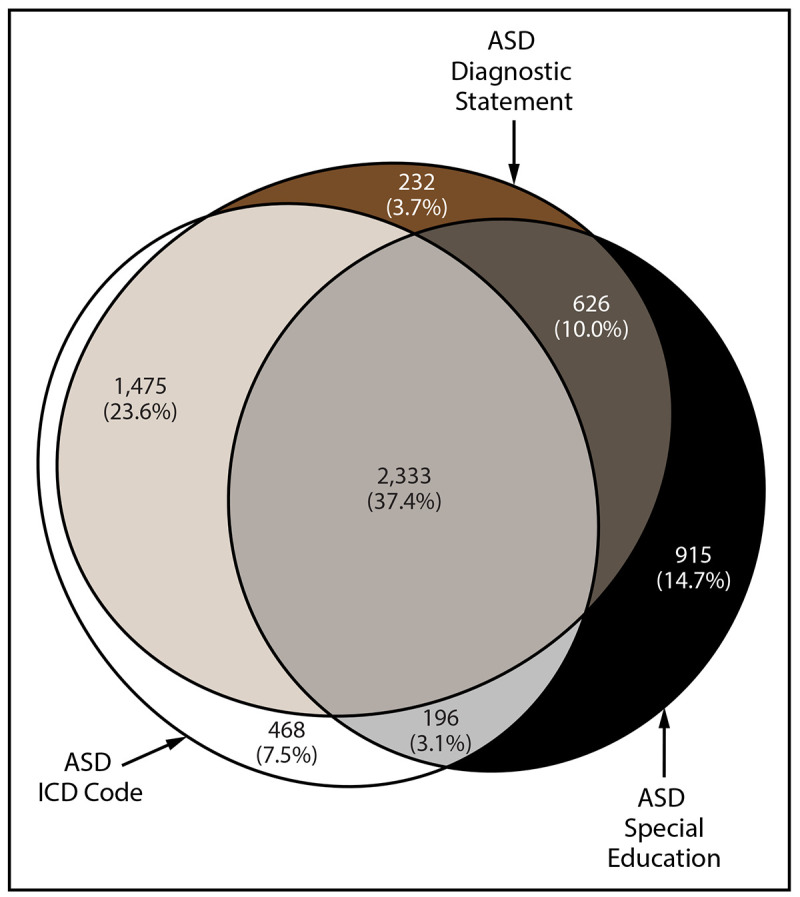
Euler diagram of different types of autism spectrum disorder identification among children aged 8 years with autism spectrum disorder* — Autism and Developmental Disabilities Monitoring Network, 11 sites, United States, 2020 **Abbreviations:** ASD = autism spectrum disorder; ICD = International Classification of Diseases. * N = 6,245.

The percentage of children with diagnostic statements, special education classifications, and ICD codes varied by site (Table 4). Across sites, the percentage of children with ASD who had a documented ASD diagnostic statement was 74.7% overall (range = 60.9% in Wisconsin to 94.7% in New Jersey). ASD prevalence per 1,000 children aged 8 years based exclusively on documented ASD diagnostic statements was 20.6 overall (range = 17.1 in Wisconsin to 35.4 in California) (Figure 2). The overall percentage of children with ASD who had a documented ASD special education classification was 65.2% (range = 44.9% in Utah to 84.9% in Minnesota) (Table 4). The percentage of children with ASD who had a documented ICD code was 71.6% (range = 51.9% in Minnesota to 82.7% in California). A majority of (74.2%) children with ASD had at least two of the three types of ASD identification documented in their records (i.e., ASD diagnostic statement, special education classification, and ASD ICD code) and 37.4% had all three (Figure 3). A majority of children with an ICD code (89.5% of 4,472 children) also had a documented ASD diagnostic statement or ASD special education classification; among all children with ASD, few (7.5% of 6,245 children) met the case definition through having an ICD code only. A majority of children with documents indicating an ASD diagnosis or ASD special education classification had these mentioned multiple times in their records (overall median number of diagnoses documented: two; range: one in Tennessee to six in New Jersey; overall median special education classifications documented: four, site-specific medians ranging from two in Wisconsin and Tennessee to six in California) (Supplementary Table 4, https://stacks.cdc.gov/view/cdc/124397).

**TABLE 4:** Autism spectrum disorder identification information among children aged 8 years meeting case definition, by site — Autism and Developmental Disabilities Monitoring Network, 11 sites, United States, 2020

Site	No. with ASD	Part of ASD case definition*			Evaluation in addition to meeting ASD case definition		
		% with ASD ICD code	% with ASD special education classification	% with ASD diagnostic statement	% with ASD with an evaluation summary diagnosis of suspected ASD	% with ASD with an evaluation summary ever ruling out ASD (diagnosis or special education classification)^†^	% with ASD ruled out (diagnosis or special education) more recently than documented ASD diagnosis or classification^†^
Arizona	360	60.8	70.8	70.8	62.8	16.4	5.8
Arkansas	362	63.0	75.7	87.8	59.7	13.0	2.5
California	710	82.7	74.5	78.9	24.5	29.3	12.8
Georgia	553	61.1	64.2	70.9	51.4	4.3	1.1
Maryland	491	60.7	74.3	83.9	70.1	13.4	2.2
Minnesota	482	51.9	84.9	63.7	8.5	6.4	1.9
Missouri	601	72.2	54.4	80.5	23.0	11.0	3.0
New Jersey	544	73.2	70.0	94.7	32.9	5.0	0.2
Tennessee	713	79.1	59.5	64.5	37.0	10.4	4.3
Utah	621	80.2	44.9	75.8	39.3	6.0	2.7
Wisconsin	808	81.4	58.4	60.9	28.0	10.5	3.3
**Total**	**6,245**	**71.6**	**65.2**	**74.7**	**37.4**	**11.6**	**3.9**

**Abbreviations:** ASD = autism spectrum disorder; ICD = International Classification of Diseases.

* ICD code, special education, and diagnosis can be interpreted as the individual sensitivity of each component related to the entire case definition.

^†^ Includes children who had ASD ruled out and never had either a documented ASD diagnosis or special education classification (i.e., had an ASD ICD code only).

Among children with ASD, 37.4% ever had an evaluation report noting that ASD was suspected but not confirmed (Table 4). Overall, 11.6% of children with ASD had an ASD diagnosis or special education eligibility ruled out (range = 4.3% in Georgia to 29.3% in California). For a majority of children, ASD was confirmed after ASD had previously been ruled out; however, 3.9% (range = 0.2% in New Jersey to 12.8% in California) of all children with ASD had an evaluation ruling out ASD more recently than one confirming ASD.

### Cognitive Ability Among Children with ASD

Data on cognitive ability were available for 4,165 (66.7%) children aged 8 years with ASD (range: 39.7% in Wisconsin to 91.2% in Arkansas) (Table 5). Among children with data on cognitive ability, the median age of the most recent cognitive test or examiner impression was 67 months (interquartile range: 51–81 months) (Supplementary Table 5, https://stacks.cdc.gov/view/cdc/124397). Girls with ASD were less likely than boys with ASD to have data on cognitive ability (64.4% versus 67.3%). Similar percentages of Black and White children had data on cognitive ability (66.8% and 65.0%, respectively), but Hispanic children (68.8%) were more likely to have cognitive data than White children. AI/AN (79.3%) and A/PI (71.2%) children and those of two or more races (73.9%) all had cognitive data at least as often as the other groups.

**TABLE 5:** Availability and distribution of IQ scores among children aged 8 years with autism spectrum disorder, by site, sex, and race and ethnicity — Autism and Developmental Disabilities Monitoring Network, 11 sites, United States, 2020

Site/Characteristic	Total no. with ASD	With IQ information	Cognitive level		
		No. (%)	IQ ≤70 (%)	IQ 71–85 (%)	IQ >85* (%)
**Site**					
Arizona	**360**	291 (80.8)	30.9	29.2	39.9
Arkansas	**362**	330 (91.2)	48.2	22.4	29.4
California	**710**	617 (86.9)	21.7	26.9	51.4
Georgia	**553**	398 (72.0)	46.2	23.6	30.2
Maryland	**491**	295 (60.1)	46.8	21.0	32.2
Minnesota	**482**	414 (85.9)	31.6	15.0	53.4
Missouri	**601**	364 (60.6)	31.9	23.6	44.5
New Jersey	**544**	342 (62.9)	38.9	30.4	30.7
Tennessee	**713**	478 (67.0)	51.0	22.0	27.0
Utah	**621**	315 (50.7)	29.2	25.4	45.4
Wisconsin	**808**	321 (39.7)	48.9	19.3	31.8
**Total**	**6,245**	**4,165 (66.7)**	**37.9**	**23.5**	**38.6**
**Sex**					
Female	**1,255**	808 (64.4)^†^	42.1^§^	21.2	36.8
Male	**4,984**	3,357 (67.3)	36.9	24.1	39.0
**Race/Ethnicity** ^¶,^ **					
AI/AN	**29**	23 (79.3)	34.8	39.1	26.1
A/PI	**476**	340 (71.4)	41.5	21.8	36.8
Black	**1,384**	925 (66.8)	50.8	25.1	24.1
Hispanic	**1,331**	916 (68.8)	34.9	27.5	37.6
White	**2,680**	1,743 (65.0)	31.8^††^	20.7	47.5
Two or more races	**261**	193 (73.9)	37.8	24.9	37.3

**Abbreviations:** AI/AN = American Indian or Alaska Native; A/PI = Asian or Pacific Islander; ASD = autism spectrum disorder.

* Includes three children stated to have an IQ score in the average range but specific score was not given.

^†^ Pearson chi-square test for proportion of males versus females with ASD and IQ information (p = 0.049).

^§^ Pearson chi-square test for proportion of males versus females with IQ ≤70 among children with ASD (p = 0.007).

^¶^ Statistically significant differences for Pearson chi-square tests for proportion of non-Hispanic Black versus non-Hispanic White children with IQ ≤70 among children with ASD (p<0.001); proportion of non-Hispanic Black versus Hispanic children with IQ ≤70 among children with ASD (p<0.001); proportion of non-Hispanic White versus non-Hispanic A/PI children with IQ ≤70 among children with ASD (p = 0.001); proportion of non-Hispanic Black versus non-Hispanic A/PI children with IQ ≤70 among children with ASD (p = 0.007).

** Persons of Hispanic origin might be of any race but are categorized as Hispanic; all racial groups are non-Hispanic.

^††^ Pearson chi-squared tests for proportion of non-Hispanic Black versus non-Hispanic White children with ASD and IQ information (p = 0.27); proportion of non–Hispanic Black versus Hispanic children with IQ information (p = 0.17); proportion of non-Hispanic White versus Hispanic children with IQ information (p = 0.03).

Among children aged 8 years with ASD who had data on cognitive ability, 37.9% were classified as having intellectual disability at their most recent test or examination, 23.5% were classified in the borderline range (IQ 71–85), and 38.6% were classified in the average or higher range (IQ >85) (Table 5). The percentage of children classified as having intellectual disability varied widely among sites (range = 21.7% in California to 51.0% in Tennessee). The median age of most recent test also varied by site (range = 55 months in Wisconsin to 79 months in Arizona) (Supplementary Table 5, https://stacks.cdc.gov/view/cdc/124397). Overall, girls with ASD were more likely to be classified as having an intellectual disability than boys with ASD (42.1% versus 36.9%), and Black children were more likely than Hispanic and White children to be classified as having intellectual disability (50.8%, 34.9%, and 31.8%, respectively). The percentage of children with ASD and intellectual disability among A/PI, two or more races, or AI/AN children was 41.3%, 37.8%, and 34.8%, respectively.

### Age at First Evaluation and ASD Diagnosis

Among 5,744 children aged 8 years with ASD and recorded evaluations, 49% were evaluated by age 36 months (range = 38.5% in Utah to 59.5% in Maryland) (Table 6). The median age at first recorded evaluation ranged from 32 months in California to 44 months in Utah. Children with ASD with an intellectual disability were more likely to be evaluated by age 36 months compared with children with ASD without an intellectual disability (61.8% versus 46.0%).

**TABLE 6:** Number and percentage of children aged 8 years with autism spectrum disorder who received a developmental evaluation by a qualified professional at age ≤36 months,* by site and intellectual disability status — Autism and Developmental Disabilities Monitoring Network, 11 sites, United States, 2020

Site	Total no. with ASD	Total with recorded evaluation			IQ ≤70			IQ >70			IQ unknown		
		No. with recorded evaluation	% evaluated by age 36 mos	Median age at earliest recorded evaluation (mos)	No. with recorded evaluation	% evaluated by age 36 mos	Median age at earliest recorded evaluation (mos)	No. with recorded evaluation	% evaluated by age 36 mos	Median age at earliest recorded evaluation (mos)	No. with recorded evaluation	% evaluated by age 36 mos	Median age at earliest recorded evaluation (mos)
Arizona	**360**	349	49.0	37	90	64.4	29	201	44.3	38	58	41.4	57
Arkansas	**362**	359	42.6	39	159	56.6	34	171	31.0	45	29	34.5	41
California	**710**	701	58.2	32	134	61.2	30	483	61.1	31	84	36.9	44
Georgia	**553**	488	46.7	39	182	51.1	36	213	46.5	40	93	38.7	42
Maryland	**491**	474	59.5	33	138	74.6	28.5	157	65.0	31	179	43.0	40
Minnesota	**482**	474	42.2	40	131	61.1	34	283	38.5	43	60	18.3	52
Missouri	**601**	591	39.8	43	116	55.2	36	245	27.3	53	230	45.2	39.5
New Jersey	**544**	537	58.3	34	133	60.9	34	208	60.6	34	196	54.1	35
Tennessee	**713**	611	43.9	41	225	61.8	31	202	41.1	43.5	184	25.0	58.5
Utah	**621**	579	38.5	44	87	48.3	39	214	30.8	49	278	41.4	42
Wisconsin	**808**	581	57.5	34	152	82.2	27	158	49.4	37.5	271	48.3	37
**Total**	**6,245**	**5,744**	**49.0**	**37**	**1,547**	**61.9**	**33.0**	**2,535**	**46.0**	**39**	**1,662**	**41.6**	**41**

**Abbreviation:** ASD = autism spectrum disorder.

* Permutation test comparing median age of earliest known evaluation for children with known IQ score ≤70 versus known IQ score >70 (p<0.001).

Among the 4,663 children aged 8 years with ASD who had an evaluation containing an ASD diagnostic statement, the median age at earliest known diagnosis was 49 months (range = 36 months in California to 59 months in Minnesota) (Table 7). Children with ASD and intellectual disability had a lower median age at diagnosis (43 months) than children without an intellectual disability (53 months). When special education classifications of autism were considered with ASD diagnoses for earliest identification, 5,579 children with ASD were identified with a median age of 52 months (range = 39 months in California and New Jersey to 60 months in Arizona).

**TABLE 7:** Median age at earliest known autism spectrum disorder diagnosis among children aged 8 years, by intellectual disability status — Autism and Developmental Disabilities Monitoring Network, 11 sites, United States, 2020

Site	Total no. with ASD	All children with an ASD diagnostic statement			Children with an ASD diagnostic statement and IQ score ≤70		Children with an ASD diagnostic statement and IQ score >70		Children with either an ASD diagnostic statement or ASD special education classification	
		No. with documented ASD diagnosis	Prevalence of ASD with documented diagnosis	Median age at earliest known diagnosis (mos)	No. with documented ASD diagnosis	Median age at earliest known diagnosis (mos)	No. with documented ASD diagnosis	Median age at earliest known diagnosis (mos)	No. with documented ASD diagnosis or ASD special education classification	Median age at earliest known ASD identification (mos)
Arizona	**360**	255	19.4	57	66	50.5	139	58	333	60
Arkansas	**362**	318	20.6	56	144	49.5	147	63	350	58
California	**710**	560	35.4	36	121	39	384	35.5	673	39
Georgia	**553**	392	17.9	50	147	48	166	52.5	476	51
Maryland	**491**	412	19.4	49	127	38.0	138	49	477	53
Minnesota	**482**	307	19.0	59	105	44	171	65	467	56
Missouri	**601**	484	19.7	51.5	99	50	194	65	556	56
New Jersey	**544**	514	27.1	38	131	37	197	39	538	39
Tennessee	**713**	458	17.9	48	192	36.5	161	56	611	58
Utah	**621**	471	19.0	56	73	52	168	65	528	58
Wisconsin	**808**	492	17.1	43	140	35.5	140	50	570	46
**Total**	**6,245**	**4,663**	**20.6**	**49**	**1,345**	43*	**2,005**	53*	**5,579**	**52**

**Abbreviation:** ASD = autism spectrum disorder.

* Permutation test comparing median age of earliest known diagnosis for children with known IQ score ≤70 versus known IQ score >70 (p<0.001).

## Discussion

For 2020, the prevalence estimate of ASD per 1,000 children aged 8 years was 27.6 (range: 23.1 in Maryland to 44.9 in California), which is higher than previous estimates from the ADDM Network. For the first time, the overall ASD prevalence for girls was >1% (11.4); in contrast, the prevalence among boys had already been noted to be higher (11.5) in the first ADDM Network report in 2002 (*4*). The continued variability in prevalence across ADDM sites, as well as the shifting in differences between demographic groups, highlight an ongoing need to better understand the systems and practices that contribute to this variability.

In its earliest years, the ADDM Network consistently reported lower overall ASD prevalence among Black and Hispanic versus White children aged 8 years. The White-Black gap in ASD prevalence narrowed in 2014, and there was no overall difference in ASD prevalence in 2016 or 2018 (Supplementary Figure 1, https://stacks.cdc.gov/view/cdc/124397). ASD prevalence among Asian, Black, and Hispanic children was at least 30% higher in 2020 than 2018, and ASD prevalence among White children was 14.6% higher than in 2018. Although this was the first time the ADDM Network reported lower ASD prevalence among White children than among other groups for children aged 8 years, a similar pattern was observed among children aged 4 years in 2018 (*18*). In addition, similar patterns were reported in analyses of national special education data and of California Developmental Services data, illustrating the prevalence of ASD classifications among Black and Hispanic children catching up and eclipsing that of White children over time (*19*,*20*). These patterns might reflect improved screening, awareness, and access to services among historically underserved groups. ASD prevalence in 2020 also was associated with lower SES, the opposite of what was observed previously (*13*), further supporting progress in identifying children regardless of race and ethnic group. As evidence grows of increased access to identification, attention might shift to what factors, such as social determinants of health, could lead to higher rates of disability among certain populations.

Even with higher ASD prevalence among Black compared with White children, Black children with ASD remained more likely to have co-occurring intellectual disability than White children, a finding that has been observed over multiple ADDM Network surveillance reports and among Black compared with White children without ASD in the United States (*21*). If Black children with ASD have less access to services than White children with ASD, as has been previously reported, the disproportionality in co-occurring intellectual disability might indicate an underascertainment of ASD among Black children without intellectual disability. Continued monitoring of trends is warranted, and it might be appropriate to re-examine potential risk or protective factors that were previously studied when the demographic composition of ASD was different.

During this period of changing demographic differences in ASD prevalence, the ADDM Network implemented two methodological changes. First, a new ASD case definition was adopted for the 2018 surveillance year. The previous case definition relied on reviewing written descriptions of ASD symptoms that were documented in comprehensive developmental evaluations. It could classify children without any formal ASD identification as ASD cases and could exclude children who had ASD diagnosed but lacked sufficient corroborating details in their records. An analysis found that non-White children were more likely to have incomplete records, which could lead to underascertainment of ASD compared with White children (*22*). However, a retroactive application of the current case definition to the 2014 and 2016 surveillance years indicates similar prevalence ratios by race and ethnicity as the previous case definition (*23*). The second change, implemented in 2020, is using population denominators with standardized racial and ethnic categories. The most important difference from the previous (bridged-race) denominators is the inclusion of a category for two or more races, which reduces the size of the denominators among the other racial groups. Nevertheless, prevalence estimates based on the previous bridged-race denominators produced a similar pattern of lower ASD prevalence among White children compared with the other groups (Supplementary Table 3, https://stacks.cdc.gov/view/cdc/124397). Thus, there were qualitatively similar patterns when consistent case definitions and denominator data sets were applied during 2014–2020.

Although ASD can be identified by age 1 year in certain cases (*24*,*25*), as described in this report, a majority of children aged 8 years living in ADDM communities were not identified until they were several years older. The reported median age of identification has not changed much over the years of ADDM Network surveillance, but it does not necessarily indicate a lack of progress in community early identification efforts. In a recent analysis of ADDM Network data during 2002–2016, the median age of diagnosis might mask progress in early detection if more children are identified (i.e., prevalence increases) at all ages and does not include children who might have ASD diagnosed after age 8 years (*26*,*27*). Therefore, the ADDM Network now reports the cumulative incidence of ASD by age 48 months as a measure of early identification and compares children aged 4 years and 8 years living in the same communities as a measure of progress (*28*,*29*). The 2020 report on early identification of ASD found more children were identified at early ages than in the past, but many are still not identified until they are school-aged (*30*).

CDC maintains a list of peer-reviewed autism prevalence studies with similar metrics to ADDM surveillance reports (https://www.cdc.gov/ncbddd/autism/data/autism-data-table.html). Other federal programs reporting ASD prevalence information in the United States include the National Survey for Children’s Health (NSCH) and the National Health Interview Survey. The ASD prevalence estimate based on the 2020 and 2021 NSCH was 2.9% and the 95% CI (2.7%–3.1%) included the 2020 ADDM Network ASD prevalence estimate (2.76%) (*31*). These surveys aim to produce nationally representative estimates among children aged 3–17 years old and ascertain information about ASD through parental report, whereas the ADDM Network estimates are not intended to be nationally representative and are generated from empirical data collected from multiple sources among participating communities. The active surveillance approach used by the ADDM Network allows reporting of when and where children are identified with ASD and affords comparisons between and within diverse U.S. communities and is not dependent on parental survey participation and ASD reporting. To facilitate comparisons between different data sources, CDC maintains an interactive website that presents U.S. state-based ASD prevalence data from four data systems (ADDM Network, NSCH, Medicaid, and special education) (https://www.cdc.gov/ncbddd/autism/data/index.html).

## Limitations

The findings in this report are subject to at least seven limitations. First, the methods rely on the availability, quality, and completeness of existing information and records to ascertain ASD cases and other indicators. Although all sites had access to special education classification data, certain sites did not have access to education records for their entire population, limiting the ability to identify children with ASD exclusively identified and served through their schools. Sites requested records from public school special education programs but did not review private school education records. Incomplete information could lead to misclassifying children’s cognitive ability, overestimating the age when they were first evaluated or when ASD was diagnosed, or failing to ascertain that the children were identified as having ASD. Sex information reflects what is represented in children’s records and might not reflect their gender identity. Second, the case definition for intellectual disability was measured using a child’s latest cognitive test or examiner statement of a child’s cognitive ability. Diagnostic and special education eligibility criteria for intellectual disability requires concurrent adaptive functioning deficits (*32*). IQ scores are not necessarily stable measures of intellectual ability over time, can increase among children with ASD in response to intensive early therapeutic interventions (*33*), and might be unstable during early childhood (*34*). The age at which children had their most recent test or examiner impression of cognitive ability varied by site. Third, the ADDM Network sites are not intended to be representative of the states in which the sites are located. ADDM Network sites are selected through an objective and competitive process, and findings do not necessarily generalize to all children aged 8 years in the United States. Interpretations of temporal trends can be complicated by changing surveillance areas, case definitions, data source access, and diagnostic practices. Fourth, small numbers result in imprecise estimates for certain sites and subgroups, and estimates falling below the selected threshold for statistical precision were suppressed. Fifth, the surveillance data system does not collect the number of ASD ICD codes a child received at a specific source, limiting comparability to analyses of claims/billing databases that consider number of ICD codes received. Sixth, the COVID–19 pandemic resulted in reduced access to records from some sources at certain sites; it was often possible to electronically obtain some data elements from these sources but not manually review the full contents of records. Disruptions in services and school closures during 2020 might have resulted in less documentation of ASD in records, which could decrease ASD ascertainment by ADDM sites. Finally, the prevalence of undetected ASD in each community as well as false-positive ASD diagnoses and classifications are unknown.

## Future Directions

For the 2022 and 2024 surveillance years, the ADDM Network will continue to monitor ASD prevalence among children aged 8 years; progress in early ASD identification among children aged 4 years; and the health status of, needs of, and planning for adolescents with ASD as they prepare to transition to adulthood. The 2020 early identification ADDM Network report documents the impact of the COVID-19 pandemic on early evaluation and detection of ASD; the effects of the pandemic on ASD identification also will be examined among children aged 4 and 8 years in future years of surveillance. Additional analyses are needed to better understand changing patterns in ASD prevalence and differences between groups; for example, changes between 2010 (when higher income was associated with higher ASD prevalence) to the present findings of higher prevalence among lower-SES neighborhoods are comparable to studies from France and Sweden (*35*,*36*). In the future, it might be possible to link the Social Vulnerability Index to children ascertained through the ADDM Network to better describe disparities within communities.

## Conclusion

Findings from the ADDM Network 2020 surveillance year indicate higher ASD prevalence than previous estimates from the ADDM Network and continuing evidence of a marked shift in the demographic composition of children identified with ASD compared with previous years. Although earlier ADDM Network reports have shown higher prevalence among higher-SES White children compared with other groups, the latest data indicate consistently higher prevalence among Black and Hispanic children compared with White children, and no consistent association between ASD and SES. Furthermore, this is the first ADDM Network report in which the prevalence of ASD among girls has exceeded 1%. Since 2000, the prevalence of ASD has increased steadily among all groups, but during 2018–2020, the increases were greater for Black and Hispanic children than for White children. These data indicate that ASD is common across all groups of children and underscore the considerable need for equitable and accessible screening, services, and supports for all children.
